# The Ecology of Withdrawal. Commentary: The NEET and Hikikomori spectrum: Assessing the risks and consequences of becoming culturally marginalized

**DOI:** 10.3389/fpsyg.2016.00764

**Published:** 2016-05-23

**Authors:** Michael E. W. Varnum, Jung Y. Kwon

**Affiliations:** Department of Psychology, Arizona State UniversityTempe, AZ, USA

**Keywords:** NEET, Hikikomori, life history theory, evolution, culture

The phenomena of NEET and Hikikomori (N/H) in Japan have attracted growing attention both by social scientists and the media. Uchida and Norasakkunkit (2015) present a novel individual difference measure meant to capture people's risk for these phenomena. Their newly developed NEET/Hikikomori Risk (NHR) scale provides a tool to assess the psychological tendencies associated with these forms of social and occupational withdrawal. Uchida and Norasakkunkit (2015) speculate about the potential causes of NHR and N/H including globalization, the importation of individualism in traditionally collectivist contexts, and changes in economic structure. Others have suggested that N/H is linked to the prolonged economic recession in Japan (Ishii and Uchida, 2016). However, the causes of N/H remain unknown. We propose that an evolutionary perspective may shed light on why in some societies a growing number of youth withdraw from social and/or occupational life in this fashion.

Throughout, most of our history as a species, withdrawing from social life and failing to actively pursue resources for extended periods of time likely dramatically reduced one's chances of survival. The fact that N/H exists at all is a testament to an incredible degree of resource abundance. Although, N/H has been most frequently observed in Japan, as Uchida and Norasakkunkit (2015) note, cases have been observed in a number of other wealthy societies (i.e., the US, UK, South Korea, Spain, Italy). We suspect that this syndrome may be more common in these societies than in societies characterized by high levels of resource scarcity. Although, systematic large scale studies comparing prevalence rates of N/H across societies have not been conducted, we would predict that such rates would be positively correlated with both GDP per capita and the degree to which social assistance policies are generous.

Uchida and Norasakkunkit (2015) also suggest that marginalized youth may be more likely to engage in deviant behaviors in societies other than Japan, whereas they may be more likely to become N/H in Japan. We would agree with this prediction but for different reasons than those outlined by the authors. In more resource scarce environments, we predict that young people who fail to gain status, resources, and friends/mates through socially approved means may be more likely to seek other paths to acquire them (such as theft, violence, prostitution, and other illegal activities) rather than withdrawing. In more desperate ecologies, ceasing to pursue such goals is more likely to jeopardize one's prospects of survival. The countries in which N/H has been documented with some frequency are all characterized by varying degrees of social safety nets and generally high levels of wealth, making withdrawing from economic and social life, and surviving for a prolonged period a feasible possibility. Thus, one might expect that higher scores on the NHR scale would be more closely linked to withdrawing from social and occupational life in richer societies, whereas higher scores on the NHR scale might be linked to engaging a range of illicit behaviors in poorer societies.

Beyond the fairly obvious observation that resources must be fairly abundant in a society for an individual to engage in these forms of withdrawal and survive, there is a deeper reason to believe resource abundance is related to N/H—namely Life History Theory (Del Giudice et al., 2015). In humans, a fast life history strategy involves a suit of tendencies including a preference for immediate rewards, impulsivity, overeating, more aggressive, and criminal behavior, and earlier and more frequent reproduction, whereas a slow life history strategy in characterized by greater investment in long term outcomes, investment in education and other types of skill acquisition, delayed reproduction, and greater parental investment (Figueredo et al., 2005, 2006; Brumbach et al., 2009; Griskevicius et al., 2011a,b; Simpson et al., 2012; Hill et al., 2016a,b). Different life history strategies are adaptive under different ecological conditions. Key ecological factors linked to life history strategies include resource scarcity (Griskevicius et al., 2011a,b), the prevalence of infectious disease (Hill et al., 2016a), and population density (Sng et al., submitted). Japan is characterized by abundant resources, relatively low prevalence of infectious disease, and high population density, all factors which push people toward slower strategies. But NEETs and Hikikomori do not appear to be pursuing slow strategies. Nor at first glance do they appear to pursue fast strategies (i.e., early reproduction or aggressive behavior). On the surface, this presents a puzzle. However, it may be the case that these individuals would pursue fast strategies if they were embedded in a context where such strategies would be adaptive or where other ecological pressures that reduce fast strategies were not present. Given a broader context (as is the case in Japan) in which these ecological pressures in addition to descriptive and injunctive norms strongly discourage fast strategies, youth who fail to achieve success in academics and/or work (i.e., those who fail at using slow strategies) may simply not have the option or the aptitude to adopt fast strategies. Thus, marginalization in a society with strong ecological pressures toward slow strategies may take the form of withdrawing from competition and interaction rather than adopting fast strategies. Withdrawal in such circumstances might be a way of conserving one's energy when one has failed at using slow strategies and fast strategies are not feasible. In other words, in circumstances when it is too costly to either continue to pursue slow strategies and when fast strategies are socially maladaptive, one might settle for securing minimal resources for somatic maintenance at minimal cost.

NEETs and Hikikomori pose a puzzle for psychologists. Why would millions of young people in a society choose to effectively withdraw from it? We believe that evolutionary psychology provides a framework to begin to understand the causes of these phenomena and why its prevalence may vary across societies.

## Author contributions

All authors listed, have made substantial, direct and intellectual contribution to the work, and approved it for publication.

### Conflict of interest statement

The authors declare that the research was conducted in the absence of any commercial or financial relationships that could be construed as a potential conflict of interest.
